# Non‐Hermitian Stealthy Hyperuniformity

**DOI:** 10.1002/advs.77054

**Published:** 2026-08-06

**Authors:** Gitae Lee, Seungmok Youn, Ikbeom Lee, Kunwoo Park, Duhwan Hwang, Seungkyun Park, Xianji Piao, Namkyoo Park, Sunkyu Yu

**Affiliations:** ^1^ Intelligent Wave Systems Laboratory, Department of Electrical and Computer Engineering Seoul National University Seoul 08826 South Korea; ^2^ Photonic Systems Laboratory Department of Electrical and Computer Engineering Seoul National University Seoul 08826 South Korea; ^3^ KAIST InnoCORE PICORE Center Korea Advanced Institute of Science and Technology Daejeon 34141 South Korea; ^4^ Wave Engineering Laboratory School of Electrical and Computer Engineering University of Seoul Seoul 02504 South Korea

**Keywords:** hyperuniformity, correlation, crystallography, microstructure, optics, rotational symmetry, scattering, parity‐time symmetry, exceptional point, structure factor

## Abstract

Symmetry‐driven wave physics in open systems, exemplified by parity‐time (PT) symmetry, has extended the landscape of crystalline phases in materials science to include gain‐loss media. Given the growing interest in engineering disorder for wave manipulation, such non‐Hermitian crystals motivate the extension of non‐Hermitian frameworks into the realm of correlated disorder. Here, we propose hyperuniformity and stealthiness in non‐Hermitian systems as a generalization of PT‐symmetric crystals to correlated disorder in the weak‐scattering limit. We extend the scattering‐microstructure correspondence to open systems, formulating non‐Hermitian hyperuniformity and stealthiness that encompass their Hermitian counterparts. This approach—incorporating a statistical crystallography framework for non‐Hermitian materials—demonstrates that real‐imaginary cross‐correlations of the material potential are irrelevant for achieving hyperuniformity but essential for characterizing stealthiness, revealing unidirectional scattering phases that are inaccessible in both Hermitian materials and non‐Hermitian crystals. Our non‐Hermitian extension also reveals unique band coalescence in the strong scattering regime, suggesting stochastic exceptional‐point dynamics. By analyzing the microstructural statistics of the resulting materials, our results—building on non‐Hermitian wave physics—establish a connection to materials science, encompassing conventional descriptors of correlated disorder.

## Graphical abstract

A framework for non‐Hermitian extensions of hyperuniformity and stealthiness is proposed, generalizing PT‐symmetric gain‐loss crystals to correlated disorder in the weak‐scattering limit. By engineering real‐imaginary correlations of the material potential, this framework enables directional scattering phases inaccessible in Hermitian materials, establishing a statistical‐crystallography approach to non‐Hermitian materials.

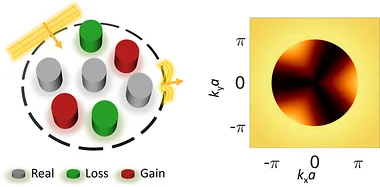

## 1 Introduction

Bridging non‐Hermitian wave physics [1, 2]—exemplified by parity‐time (PT) symmetry [3]—with discrete translational symmetry has rejuvenated crystallography by extending traditional band theory to systems composed of periodically arranged gain and loss media. This generalized band theory is founded on concepts underlying open‐system wave phenomena: biorthogonal bulk‐boundary correspondence [4], nontrivial topology around exceptional points (EP) [5, 6], and generalized Brillouin zones [7]. Building on these well‐established analytical tools, the traditional realm of crystals has been extended into non‐Hermitian regimes, unveiling exotic wave phenomena in PT‐symmetric crystals, such as non‐Hermitian skin effects [8], unidirectional transparency [9] and modal conversion [10], and non‐Abelian band braiding [11].

In classifying material phases according to their microstructures, the regime of order has been substantially extended through the concepts of hyperuniformity (HU) [12, 13] and stealthiness [14]. Originally developed in microstructural statistics to describe the vanishing of long‐wavelength density fluctuations [13, 15], these concepts exhibit an intriguing correspondence with wave phenomena [16]—the suppression of long‐wavelength scattering over a range of reciprocal space in the weak‐scattering regime. This correspondence has revealed numerous previously unrecognized wave phenomena, including perfect isotropic bandgaps [17, 18, 19, 20] and transparency [21], symmetry‐free guiding and resonances [22, 23], the screening of material microstructures [24], and patternless spectral filtering [25]. Given the extension from traditional Hermitian crystals to PT‐symmetric crystals from a wave‐physics perspective, and the interpretation of HU and stealthiness as statistically generalized order, a natural question arises: what is the non‐Hermitian generalization of HU and stealthiness? To address this question, we can envisage the use of the correspondence between microstructural statistics and weak‐scattering phenomena in open systems, in line with previous studies of Hermitian systems.

Here, we propose non‐Hermitian generalizations of hyperuniformity (HU) and stealthiness by leveraging the scattering‐microstructure correspondence. By characterizing the conditions for suppressed scattering in weakly perturbed non‐Hermitian materials, we define HU and stealthiness under non‐Hermiticity. This approach reveals that cross‐correlations between the real and imaginary parts of the material potential are critical only for stealthiness. We develop a statistical crystallography framework to classify non‐Hermitian stealthiness by exploiting the rotational symmetries of these correlations, thereby unveiling novel material phases with unidirectional scattering responses. To connect these wave‐physics‐based concepts to established material phases, we also examine the microstructural statistics of non‐Hermitian HU and stealthy HU (SHU). Although our definitions are rooted in the weak‐scattering limit, band coalescence governed by correlation symmetries in our SHU configurations suggests that the framework remains relevant to non‐Hermitian band theory for disordered materials. Our results, extending correlated disorder to open systems, provide new design freedom for wave functionality, particularly through non‐Hermiticity‐induced directionality.

## 2 Results

### 2.1 Non‐Hermitian HU

**FIGURE 1:**
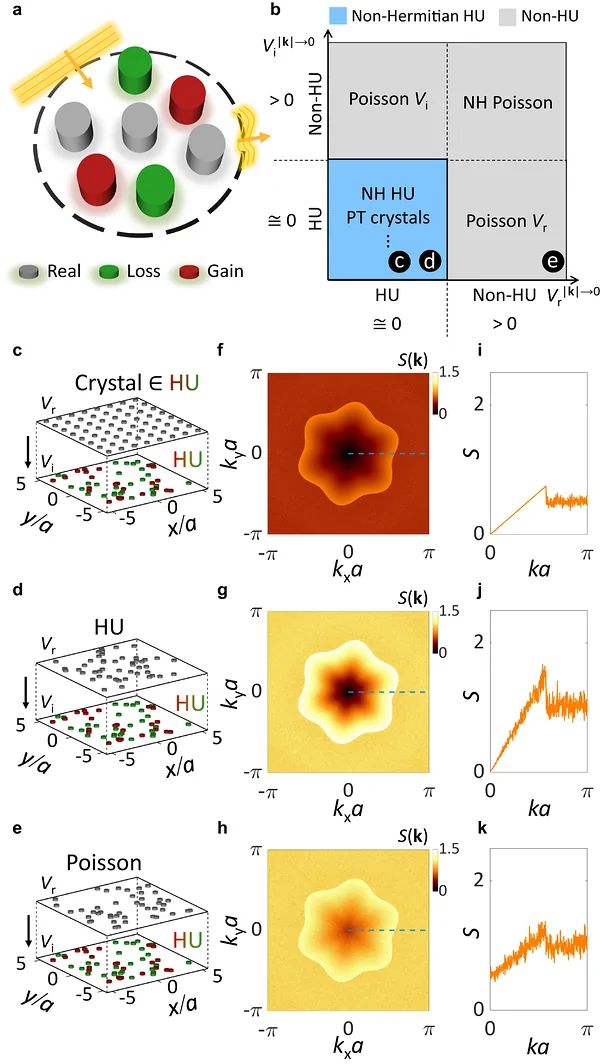
Non‐Hermitian HU. a) Schematic illustrating scattering from a multiparticle non‐Hermitian material. Gray, red, and green rods represent real‐valued, purely gain, and purely loss scatterers, respectively. b) Phase diagram characterizing non‐Hermitian HU. The phases are classified with *V*_r_(|**k**| → 0) and *V*_i_(|**k**| → 0); ‘NH HU’ denotes non‐Hermitian HU; ‘PT’ represents PT symmetry; ‘Poisson *V*_r_’ and ‘Poisson *V*_i_’ represent real and imaginary Poisson potentials, while preserving *V*_i_(|**k**| → 0) ≅ 0 and *V*_r_(|**k**| → 0) ≅ 0, respectively; ‘NH Poisson’ denotes the Poisson distributions for both *V*_r_ and *V*_i_. c–e) Examples of *N*‐particle non‐Hermitian materials: ‘NH HU’ materials (c,d) and ‘Poisson *V*_r_’ (e). f–k) The corresponding structure factors (f–h) and their cross‐sections ((i–k); blue dashed lines in (f–h)). *a* = *L*/*N* ^1/2^ for a square supercell of side length *L* in (c–k).

In Hermitian systems, a HU material with suppressed long‐wavelength density fluctuations corresponds to scattering suppression near zero momentum shift, |**k**| ≅ 0, under the first‐order Born approximation [12, 13, 16]. Motivated by this correspondence, we extend HU to non‐Hermitian wave physics in the weak‐scattering regime by analyzing the conditions for scattering suppression in a nonconservative composite material containing gain and loss media (Figure 1a). We begin with the wave equation ∇^2^ *ψ* + *V*(**r**)*ψ* = 0 for a complex‐valued potential *V*(**r**). To formulate scattering problems, we set *V*(**r**) to be spatially confined to a finite domain Ω: *V*(**r**∈Ω) = *V*_o_ + *V*_a_(**r**) and *V*(**r**∈Ω^c^) = *V*_o_, where *V*_o_ > 0 is a constant, real‐valued bias potential and *V*_a_(**r**) is a spatially varying, complex‐valued potential. For the planewave incidence *ψ*_in_(**r**) = *ψ*_I_exp(*i* **k**_I_∙**r**) with |**k**_I_|^2^ = *V*_o_, the Lippmann‐Schwinger equation becomes $\psi \left(R\right)=\psi_{\mathrm{in}}\left(R\right)+\frac{1}{4\pi }\int G\left(R,r;k_{S}\right)V_{a}\left(r\right)\psi \left(r\right)dr$, where *G*(**R**,**r**;**k**_S_) is the Green's function defined by ∇^2^ *G*(**R**,**r**;**k**_S_) + *V*_o_ *G*(**R**,**r**;**k**_S_) = –4*πδ*(**R**—**r**) and **k**_S_ denotes the scattering wavevector [26]. Under the Born approximation, the far‐field scattering intensity is well approximated as *I*_S_(**R**) ∝ *S*(**k** ≜ **k_S_**—**k_I_**)|*ψ*_I_|^2^ using the structure factor *S*(**k**) = *S*_A_(**k**) + *S*_C_(**k**), defined as follows (Supporting Information: Note S1):

(1) SA(k)=1VΩVr(k)2+Vi(k)2,SC(k)=2VΩImVr(k)Vi*(k),

where *V*_r_(**k**) and *V*_i_(**k**) are the reciprocal‐space potentials of the real and imaginary parts of *V*_a_(**r**), respectively, *V*_Ω_ is the volume of Ω, and 〈…〉 denotes the statistical average under the ergodic condition [27]. Notably, nonconservative perturbations with Im[*V*_a_(**r**)] ≠ 0 contribute to scattering through the auto‐ and cross‐correlations, 〈|*V*_i_(**k**)|^2^〉 and 〈*V*_r_(**k**)*V*_i_^*^(**k**)〉, respectively.

We emphasize that scattering from a non‐Hermitian material is constrained by the conjugate symmetries of the complex‐valued potentials in reciprocal space: *V*_r_(–**k**) = *V*_r_^*^(**k**) and *V*_i_(–**k**) = *V*_i_^*^(**k**). Applying these symmetries to Equation (1) yields contrasting parity conditions under inversion: *S*_A_(–**k**) = *S*_A_(**k**) and *S*_C_(–**k**) = –*S*_C_(**k**), which is analogous to the PT‐symmetric condition of the real and imaginary parts of the potential [1, 2, 3]. Applying the scattering‐suppression criterion for HU, the non‐Hermitian generalization of HU requires the following condition:

(2) $$\lim_{\left|k\right|\to 0}\left[S_{A}\left(k\right)+S_{C}\left(k\right)\right]=\lim_{\left|k\right|\to 0}S_{A}\left(k\right)=0,$$

because |*S*_A_(**k**)| ≥ |*S*_C_(**k**)| for all **k**. Therefore, Equations (1) and (2) lead to the definition of non‐Hermitian HU (Figure 1b)—simultaneous HU of the real and imaginary parts of the potential *V*_a_(**r**), such that |*V*_r_(**k**)|^2^→0 and |*V*_i_(**k**)|^2^→0 as |**k**|→0, remarkably, regardless of their relative spatial distributions.

To elucidate this definition of non‐Hermitian HU, we present a variety of point‐particle material microstructures in Figure 1c–e (see ‘Non‐Hermitian scatterers’ in Section 4) together with their structure‐factor profiles (Figure 1f–k), which comprise real‐valued scatterers (Im[*V*_a_(**r**)] = 0) and imaginary‐valued gain‐loss scatterers (Re[*V*_a_(**r**)] = 0). When HU features are imposed on both the real and imaginary particles (Figure 1c,d), long‐wavelength scattering is successfully suppressed with *I*_S_(**R**) ≅ *S*(**k**→0) ≅ 0 (Figure 1f,g,i,j), in sharp contrast to the case where a non‐HU configuration is present in either the real or imaginary particle profile (Figure 1e,h,k). This observation demonstrates that non‐Hermitian systems provide extended degrees of freedom for altering the scattering features (Figure 1f,g) via distinct microstructures of the real and imaginary HU potentials and their cross‐correlation (Figure 1c,d), while strictly preserving HU suppression of long‐wavelength scattering.

### 2.2 Density Fluctuations of Non‐Hermitian HU

To confirm the validity of the proposed non‐Hermitian HU, the resulting material microstructures should allow a rational interpretation from a materials‐science perspective, especially encompassing the traditional definition of HU in Hermitian systems. We therefore examine density fluctuations in non‐Hermitian materials across different material phases, focusing on the uniqueness of non‐Hermitian HU. In Hermitian systems, HU microstructural statistics manifest as suppressed number‐density fluctuations, such as *σ* ^2^(*R*)/*R^d^* ∼ 1/*R*, in sharp contrast to the constant *σ* ^2^(*R*)/*R^d^* observed in uncorrelated disorder [13], where *R* is the radius of the sampling window used to measure number‐density fluctuations and *d* denotes the system dimensionality. For a complex‐valued potential *V*_a_(**r**), this density‐based analysis must be generalized into three quantities (see ‘Density fluctuations’ in Section 4): the auto‐density fluctuations, *σ*_AR_^2^(*R*)/*R^d^* and *σ*_AI_^2^(*R*)/*R^d^*, which denote the volume‐normalized variances of Re[*V*_a_(**r**)] and Im[*V*_a_(**r**)], respectively, and the cross‐density fluctuation, *σ*_C_^2^(*R*)/*R^d^*, which is the volume‐normalized covariance between Re[*V*_a_(**r**)] and Im[*V*_a_(**r**)].

**FIGURE 2:**
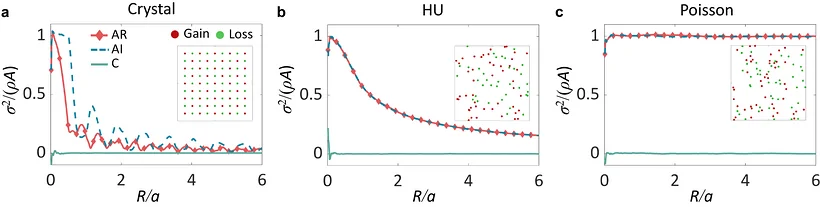
Density fluctuations in non‐Hermitian materials. a–c) Density‐fluctuations in a PT‐symmetric square‐lattice crystal (a), a disordered non‐Hermitian HU (b), and an uncorrelated non‐Hermitian Poisson material (c). Each inset shows the portion of representative material realizations. Coordinates and window radius are normalized by *a* = *L*/*N* ^1/2^. Variances are calculated over an ensemble of 100 realizations. The cinnabar‐marker and blue‐dashed curves denote the volume‐normalized variances of the real and imaginary potentials, respectively, while the teal‐solid curve represents the volume‐normalized covariances between them.

Figure 2 examines our wave‐physics‐based definition of non‐Hermitian HU through a microstructural analysis (see ‘Density measurements’ in Section 4; Supporting Information: Note S2 for semi‐analytical demonstration). For each realization, we sample the potential *V*_a_(**r**) of an *N*‐particle material using a circular window of radius *R* to compute the normalized density‐fluctuation measures, *σ*_AR_^2^(*R*)/(*ρA*), *σ*_AI_^2^(*R*)/(*ρA*), and *σ*_C_^2^(*R*)/(*ρA*), where *ρ* is the number density of scatterers and *A*(*R*) = *πR* ^2^ is the window area (see Supporting Information: Note S2 for analytical expressions in terms of pair correlation functions). We compare different types of non‐Hermitian HU materials—a PT‐symmetric crystal (Figure 2a) and a disordered non‐Hermitian HU material (Figure 2b)—with a non‐Hermitian Poisson material defined in Figure 1b (Figure 2c).

The results demonstrate that both the PT‐symmetric crystal and the disordered non‐Hermitian HU material exhibit suppressed auto‐density fluctuations as *R* increases (Figure 2a,b), which underlies the long‐wavelength scattering suppression *S*(|**k**| → 0) = 0. In contrast, the Poisson configuration exhibits *R*‐independent plateaus in the auto‐density fluctuations (Figure 2c), a hallmark of uncorrelated disorder. These results confirm that non‐Hermitian HU preserves the conventional scaling behavior of density fluctuations for the real and imaginary parts of the potential separately. Notably, the near‐zero cross‐density fluctuations observed in our configurations originate from the balanced numbers of gain and loss particles and their matched statistical distributions, remaining further design freedom to alter microstructures while preserving HU (Supporting Information: Note S2).

### 2.3 Non‐Hermitian SHU

Beyond the non‐Hermitian generalization of HU, we explore a non‐Hermitian generalization of stealthiness [14], which is defined by *S*(|**k**| ≤ *K*) ≈ 0 in Hermitian systems for a finite positive scalar *K*. We note that suppression of scattering over |**k**| ≤ *K* is governed by inversion symmetries, *S*_A_(–**k**) = *S*_A_(**k**) and *S*_C_(–**k**) = –*S*_C_(**k**). In contrast to non‐Hermitian HU, these symmetries impose angular constraints on realizing non‐Hermitian SHU. To characterize these constraints, we develop a statistical crystallography framework for classifying non‐Hermitian correlated disorder by examining the rotational symmetries of statistical metrics relevant to long‐wavelength scattering.

**FIGURE 3:**
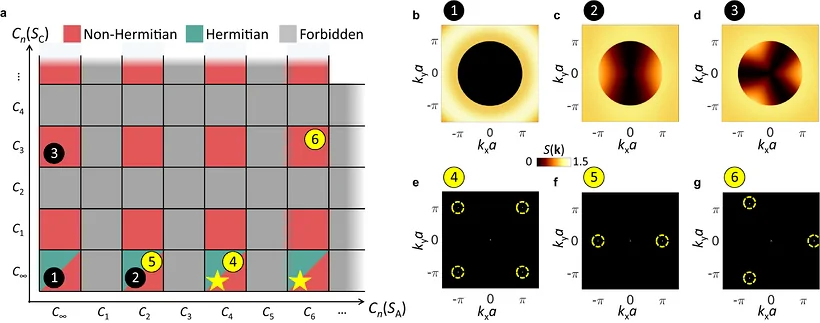
Statistical crystallography for non‐Hermitian order and SHU. a) Classification of scattering responses by the rotational symmetries of *S*_A_(**k**) and *S*_C_(**k**). Cinnabar and teal colors denote scattering responses that can be realized in non‐Hermitian and Hermitian materials, respectively. Yellow stars denote 2D Hermitian crystals with regular polygon tiling. ‘Forbidden’ indicates inaccessible scattering responses due to inherent constraints on *S*_A_(**k**) and *S*_C_(**k**). b–d) Non‐Hermitian SHU with *C*_∞_ (b), *C*_2_ (c), and *C*_3_ (d) symmetries. e–g) PT‐symmetric crystals with *C*_4_ (e), *C*_2_ (f), and *C*_3_ (g) symmetries. All materials consist of 10^6^ scatterers. The structure factor is obtained through the average of 10^2^ ensemble realizations.

In our framework, we examine the rotational symmetries encoded in the angular structure of the constraints *S*_A_(|**k**| ≤ *K*) ≈ 0 and *S*_C_(|**k**| ≤ *K*) ≈ 0 (Figure 3a)—including the symmetries of the Bragg peaks at |**k**| ≈ *K* in crystalline structures. Conventional Hermitian materials (boxes shaded in teal in Figure 3a), which always satisfy *S*_C_(**k**) = 0, span a parameter range in which the allowed symmetry classes of *S*_A_(**k**) are *C*_2_ *_m_*, while those of *S*_C_(**k**) are *C*_∞_, where *C_n_* denotes the group of *n*‐fold rotational symmetry. Although square, triangular, and honeycomb lattices—representing the 2D crystals accessible via regular polygon tiling—occupy only a subset of this range (yellow stars in Figure 3a), SHU spans a broader set of symmetry classes, including all *C*_2_ *_m_* symmetries attainable with isotropic [13] and anisotropic SHU [28] materials.

In contrast, we emphasize that non‐Hermitian configurations with a nonzero cross‐correlation *S*_C_(**k**)—which exhibits inherent *C*_2_ *_m_*_+1_ symmetries—substantially extend the accessible range of scattering responses (boxes shaded in cinnabar in Figure 3a). In Figure 3b–g, we illustrate the structure factors *S*(**k**) of a variety of non‐Hermitian ordered and disordered structures, including disordered SHU phases inversely designed using ansatz functions [29, 30] (Figure 3b–d; see ‘Non‐Hermitian SHU ansatz’ and ‘Inverse design’ in Section 4 and Supporting Information: Note S3) and examples of PT‐symmetric crystals (Figure 3e–g; Supporting Information: Notes S4 and S5 for extended analysis with full‐wave simulations [31]). Notably, non‐Hermitian materials can exhibit long‐wavelength scattering phases identical to those obtained with Hermitian systems: conventional SHU (Figure 3b), anisotropic SHU (Figure 3c) [28], a *C*_4_‐crystal (Figure 3e), and an anisotropy‐induced *C*_2_‐crystal (Figure 3f). Such isoscattering responses in the region |**k**| ≤ *K* provide design freedom to tailor other wave characteristics, such as scattering at |**k**| > *K* or the spectral bandwidth [16, 24].

More importantly, non‐Hermitian degrees of freedom enable rotational‐symmetry classes in long‐wavelength scattering that are inaccessible in Hermitian systems—for example, *C*_3_‐crystals (Figure 3g), and *C*_3_ disordered SHU materials (Figure 3d), which satisfy *S*(**k**) ≠ *S*(–**k**) and can even support unidirectional scattering. This result—while remaining consistent with electromagnetic reciprocity in the absence of time‐reversal symmetry breaking [32]—identifies the microstructural conditions in non‐Hermitian correlated disorder that lead to asymmetric scattering, a phenomenon that has been intensively studied in PT‐symmetric crystals [1].

We note that the angular symmetries of the scattering observed in Figure 3 highlight the necessity of interpreting the SHU condition for non‐Hermitian materials in terms of angularly restricted, lower‐dimensional configurations. Remarkably, because *S*_C_(**k**) enlarges the space of possible angular responses in the overall scattering, it is useful to consider a 1D SHU condition of the form of *S*(|**k**| ≤ *K*; *θ*(**k**) ∈ Φ) ≈ 0 in our 2D example, where *θ*(**k**) is the polar angle of **k** and Φ denotes the set of angles over which the SHU condition is satisfied. In this formulation, Φ is determined by the *C_n_* symmetries of *S*_A_(**k**) and *S*_C_(**k**), whereas the conventional 2D SHU—corresponding to the full angular range of Φ = [0, 2*π*)—is recovered when both *S*_A_(**k**) and *S*_C_(**k**) belong to *C_∞_*.

### 2.4 Density Fluctuations of Non‐Hermitian SHU

**FIGURE 4:**
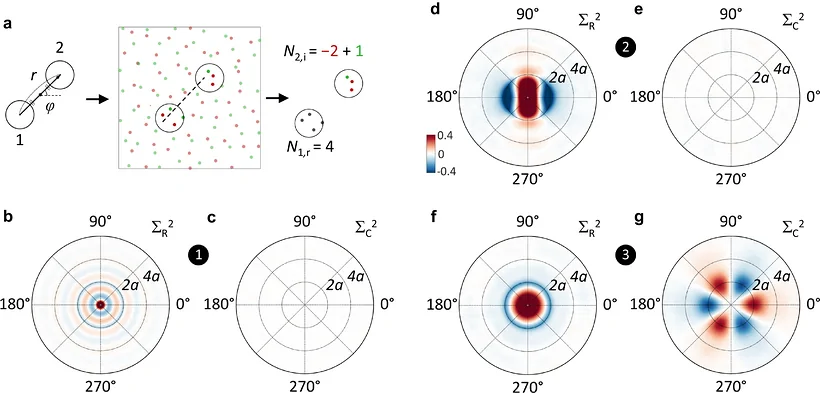
Directional number statistics. a) Extraction of directional number statistics using two circular, correlated windows with fixed radii of characteristic length *a*. The displacement between two windows is swept with parameters *r* and *φ*. Window pairs are placed uniformly at random in the unit cell for 10^5^ trials for each (*r*, *φ*). b–g) Auto‐ and cross‐covariances of non‐Hermitian SHU under *C*_∞_ (b,c), *C*_2_ (d,e), and *C*_3_ (f,g) symmetries described in Figure 3. Radial distance and azimuthal angle of polar plots correspond to *r* and *φ*, respectively.

Along with the analysis in Figure 2, we examine the material microstructures of the obtained non‐Hermitian SHU, thereby connecting our definition to a material‐science interpretation. To capture angularly varying density fluctuations in non‐Hermitian multiphase materials, we generalize the conventional number‐variance measure by using two correlated observation windows 1 and 2 with the same radius *a* (Figure 4a and Supporting Information: Note S6), which is sufficiently small to probe local distributions. The displacement from the center of window 1 to that of window 2 is expressed in polar coordinates (*r*, *φ*). Using the windows, we measure the integrated amount of real and imaginary parts of the potential perturbation within window *n* (=1, 2), denoted as *N_n_*_,r_ and *N_n_*_,i_, respectively. Pairs of windows are placed uniformly at random throughout the unit cell of the material for 10^5^ trials, to obtain stable statistics of (*N*_1,r_, *N*_1,i_, *N*_2,r_, *N*_2,i_).

Figure 4b–g shows the microstructural statistics of non‐Hermitian disordered SHUs in Figure 3b–d, for their auto‐ (Σ_A_^2^ = ⟨*N*_1,r_ *N*_2,r_⟩ + ⟨*N*_1,i_ *N*_2,i_⟩; Figure 4b,d,f) and cross‐ (Σ_C_^2^ = ⟨*N*_1,i_ *N*_2,r_⟩ − ⟨*N*_1,r_ *N*_2,i_⟩; Figure 4c,e,g) covariances of real and imaginary potentials. Notably, the structure factors accessible in Hermitian systems (Figure 3b,c) correspond to the complete suppression of real‐imaginary covariances Σ_C_^2^ (Figure 4c,e), as expected from the vanishing scattering interference with *S*_C_(**k**). On the other hand, the unidirectional scattering response in Figure 3d corresponds to the emergence of Σ_C_^2^ (Figure 4g). We note that the emergence of the characteristic length ∼2*a* and its antisymmetric profile of Σ_C_^2^ indicate that our non‐Hermitian disordered SHUs correspond to a disordered composite of PT‐symmetric dipoles.

The proposed approach for capturing microstructural features of non‐Hermitian materials reduces to a coarse‐grained representation of the conventional two‐point correlation function in the limit of a vanishing window size. However, the method differs conceptually and practically by probing finite‐support correlations at a prescribed mesoscopic scale. By integrating local quantities over observation windows, the method is robust against pointwise noise, sharp interfaces, and phase discontinuities, and is well suited for both numerical simulations and experimental measurements of non‐Hermitian materials. Importantly, explicit control of the window size enables the characterization of angularly varying features at a target mesoscopic length scale, complementing the information obtained from pointwise correlation analysis.

### 2.5 Non‐Hermitian Bands Beyond Weak Scattering

**FIGURE 5:**
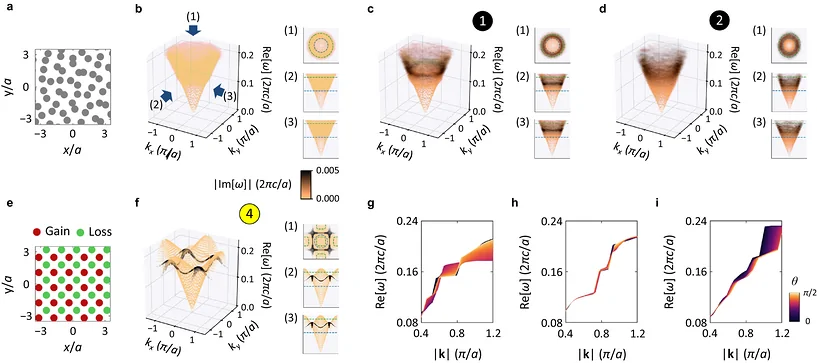
Non‐Hermitian band coalescences. a,b) Hermitian SHU of rods with *ε* = 12 (a) and its unfolded stochastic band structure (b). c,d) Band structures of isotropic (*C*_∞_) (c) and anisotropic (*C*_2_) (d) non‐Hermitian SHU with balanced gain and loss. e,f) PT‐symmetric crystal (e) and its band structure (f). Colors in (b‐d) and (f) denote |Im[*ω*]| of each (k, Re[*ω*]) state. The encircled numbers in (c), (d), and (f) correspond to the phases in Figure 3, respectively. In each of (b–d) and (f), insets (1)–(3) show orthographic projections of the band structure taken along the viewing directions indicated by the arrows in (b): (1) is the top view along the Re[*ω*] axis, while (2) and (3) are side views along the two orthogonal in‐plane directions. The blue (Re[*ω*] = 0.12 × (2*πc*/*a*)) and green (Re[*ω*] = 0.20 × (2*πc*/*a*)) dashed curves in inset (1) mark two representative IFCs, whose ellipticities are 0.009 and 0.032 (b), 0.001 and 0.047 (c), and 0.066 and 0.096 (d); the near‐zero value in (c) reflects the isotropy of the *C*_∞_ SHU, whereas the larger values in (d) quantify the anisotropy of the *C*_2_ SHU. *ε* = 12 ± 1.5*i* in (c–f). g–i) Angle‐resolved dispersions Re[*ω*] versus |**k**| for the crystal (g), isotropic SHU (h), and anisotropic SHU (i); colors denote the momentum angle *θ*. Each stochastic band is calculated using 10 random material realizations.

Our definitions of non‐Hermitian HU and stealthiness, together with the validity of the corresponding scattering responses discussed so far, rely on the weak‐scattering regime. However, the unique microstructures of these materials shown in Figures 2 and 4 motivate further exploration of the richness of non‐Hermitian physics beyond weak scattering, such as PT‐symmetry breaking and EPs emerging in band theory. As an initial testbed, we examine how the band coalescence observed in PT‐symmetric crystals [33] manifests in our non‐Hermitian SHU materials. Because of broken discrete translational symmetry, we estimate the dispersion bands of non‐Hermitian SHU stochastically using the band unfolding method [34, 35, 36] (see ‘Stochastic band estimation’ in Section 4). For comparison with PT‐symmetric crystals [33], we employ cylindrical rods with the relative permittivities of Re[*ε*] = 12 and |Im[*ε*]| ≤ 1.5, embedded in air, which lie in the strong‐scattering regime. Figure 5a,b shows a Hermitian SHU structure and its unfolded dispersion band, respectively, exhibiting an isotropic, cone‐like geometry.

Figure 5c,d shows the stochastic bands for isotropic (*C*_∞_) and anisotropic (*C*_2_) non‐Hermitian SHU materials, respectively, which are compared with a PT‐symmetric crystal (Figure 5e) and its band structure (Figure 5f); the color encodes the imaginary eigenfrequency |Im[*ω*]| of each state. Notably, both the isotropic SHU (Figure 5c) and the crystal (Figure 5f) develop a well‐defined maximum |Im[*ω*]| across the Re[*ω*]‐axis—the hallmark of PT‐symmetry breaking through band coalescence [33]—demonstrating that EP dynamics can emerge stochastically in non‐Hermitian SHU. Remarkably, the coalescence in the SHU is angularly isotropic, in contrast to the discrete rotational symmetry of the crystal. By contrast, the anisotropic SHU exhibits an ill‐defined maximum |Im[*ω*]| and elliptic isofrequency contours (IFCs) (Figure 5d), reflecting broken 2D SHU and anisotropy in the structure factor (Figure 3c) due to incomplete suppression of long‐wavelength density fluctuations along certain directions.

This contrast also appears in the Re[*ω*] dispersion resolved by the **k**‐space polar angle *θ* (Figure 5g–i). As examined in the previous study [33], the PT‐symmetric crystal exhibits flat, coalescence‐induced spectral regions that are strongly anisotropic (Figure 5g), whereas the isotropic non‐Hermitian SHU develops comparable flat regions with almost angle‐independent trajectories (Figure 5h). The anisotropic SHU shows less pronounced flattening but a clear angular spread (Figure 5i). These results demonstrate that the statistical order encoded in the non‐Hermitian structure factor persists as band‐level phenomena beyond the weak‐scattering regime, extending PT‐symmetric band coalescence from crystals to correlated disorder.

## 3 Discussion

Although our disordered non‐Hermitian HU systems correspond to statistical generalizations of PT‐symmetric crystals in the weak‐scattering limit, the results suggest a route toward extending non‐Hermitian band theory into the regime of correlated disorder, as exemplified in the band coalescence example. Analogous to the extension of topological physics to HU materials [37], we can map the point‐process realization of gain and loss scatterers onto the positions of discretized elements in a tight‐binding description. This approach provides a basis for more rigorous future extensions of phenomena from non‐Hermitian band theory to non‐Hermitian SHU systems. For example, the anisotropy of exceptional rings that is unavoidable in non‐Hermitian crystals [33, 38] can be replaced by perfectly isotropic rings in non‐Hermitian SHU, as demonstrated in Figure 5c,h. Likewise, exploring various phenomena, such as EP‐related topology [5, 6] and non‐Hermitian skin effects [8] in non‐Hermitian SHU, can be considered potential research topics. Further studies in directions related to topological Anderson insulators [39] may provide a useful direction for exploring correlated features in SHU, particularly in relation to wave localization in non‐Hermitian media [40].

In terms of a practical implementation, our study relies on the Born approximation in the weak‐scattering regime, which is well suited to dilute or low‐contrast non‐Hermitian media (see Supporting Information: Note S7). Considering the challenge in handling gain scatterers, one can employ passive demonstrations based on a gauge transformation [41] at the cost of the overall scattering intensity (see Supporting Information: Note S8) or use an amplifying environment with modified Green's functions, which includes subwavelength lossy scatterers [42]. By conceptualizing gain and loss scatterers as distinct material phases, our work also extends the framework of SHU in multiphase media incorporating open‐system design freedom, which holds potential for multiphysics applications [43, 44, 45].

In summary, we generalize HU and stealthiness to non‐Hermitian wave systems by establishing a wave‐based definition grounded in scattering suppression and microstructural statistics. Within the weak‐scattering regime, our framework shows that non‐Hermitian HU requires independent suppression of long‐wavelength fluctuations in both the real and imaginary parts of the potential, while non‐Hermitian SHU depends crucially on their cross‐correlations. Building on the rotation‐symmetry‐based crystallography developed here, we demonstrate directional suppression of scattering in disordered non‐Hermitian media. More broadly, by bridging non‐Hermitian wave physics and material microstructures, our results, including isotropic band coalescences, suggest a route toward extending PT‐symmetric concepts beyond periodic lattices, paving the way toward directional wave manipulations in optics, acoustics, and electronic circuits.

## 4 Methods

### 4.1 Non‐Hermitian Scatterers

In our analysis, we employ the point‐particle approximation, which yields the structure factor for an *N*‐particle material:

(3) $$S\left(k\right)=\frac{1}{\sum_{p=1}^{N}{\left|f_{p}\right|}^{2}}{\left|C(k)\right|}^{2},$$

where *C*(**k**) ≜ ∑*_p_ f_p_* exp(−*i* **k**·**r** *_p_*) represents the collective coordinate variable, and **r** *_p_* and *f_p_* = *f*_r,_ *_p_* + *if*_i,_ *_p_* denote the position and complex‐valued scattering form factor of the *p*th scatterer, respectively, with *f*_r,_ *_p_*, *f*_i,_ *_p_* ∈ ℝ. For the results in Figure 1, we set *f_p_* ∈ {1, −*i*, +*i*}, while we use *f_p_* ∈ {1 − *i*, 1 + *i*} in Figures 2, 3, 4 except for Figure 3g with *f_p_* ∈ {1 – *i*/3^1/2^, 1 + *i*/3^1/2^} for maximized unidirectionality. Especially, we can express the form‐factor components as *f*_r,_ *_p_* = *f*_r_ and *f*_i,_ *_p_* = *s_p_f*_i_, by setting *s_p_* ∈ {−1, +1} in Figures 2, 3, 4 and *f*_i_ = 1/3^1/2^in Figure 3g. The applied condition ensures that the contributions of auto‐ and cross‐correlations to scattering are comparable in magnitude.

### 4.2 Density Fluctuations

For the analysis in Figure 2, we introduce the indicator function *w*(**r**—**x**_0_; *R*) associated with a circular observation window of radius *R* centered at **x**_0_. This function is defined as *w*(**r**—**x**_0_; *R*) = 1 if a scatterer is located within the window and *w*(**r**—**x**_0_; *R*) = 0 otherwise. Using this indicator function, the complex‐valued potential perturbation from scatterers within the window is expressed as *N*_c_ = *N*_r_ + *iN*_i_, where

(4) $$N_{r}\left(x_{0};R\right)=\sum_{p}f_{r}w\left(r_{p}-x_{0};R\right),N_{i}\left(x_{0};R\right)=\sum_{p}s_{p}f_{i}w\left(r_{p}-x_{0};R\right).$$

The normalized density fluctuation—defined as the variance of the potential perturbation normalized by the total perturbation—is generalized as

(5) $$\frac{\langle {N_{c}}^{2}\rangle -{\langle N_{c}\rangle }^{2}}{\rho A(R)}=\frac{{\sigma^{2}}_{\mathrm{AR}}\left(R\right)}{\rho A(R)}-\frac{{\sigma^{2}}_{\mathrm{AI}}\left(R\right)}{\rho A(R)}+2i\frac{{\sigma^{2}}_{C}\left(R\right)}{\rho A(R)},$$

for the variance and covariance expressions:

(6) $$\begin{array}{c} {\sigma_{\mathrm{AR}}}^{2}\left(R\right)=\mathrm{cov}\left[N_{r}\left(x_{0};R\right),N_{r}\left(x_{0};R\right)\right],\\ {\sigma_{\mathrm{AI}}}^{2}\left(R\right)=\mathrm{cov}\left[N_{i}\left(x_{0};R\right),N_{i}\left(x_{0};R\right)\right],\\ {\sigma_{C}}^{2}\left(R\right)=\mathrm{cov}\left[N_{r}\left(x_{0};R\right),N_{i}\left(x_{0};R\right)\right], \end{array}$$

where cov[*a*,*b*] denotes the covariance between the random variables *a* and *b* over an ensemble of random window positions **x**_0_.

### 4.3 Density Measurements

In our material configuration, point scatterers are distributed within a square supercell of side length *L* under periodic boundary conditions, with the number density of *ρ* = *N*/*L* ^2^ and the characteristic length of *a* = 1/*ρ* ^1/2^. Auto‐ and cross‐densities are estimated using the Monte Carlo window sampling [13]. For a given window center **x**_0_ and radius *R*, we compute the sums of the potential perturbations inside the window using Equation (4). Using the obtained *N*_r_ and *N*_i_, we numerically calculate the auto‐ and cross‐densities by estimating their variances and covariance.

### 4.4 Non‐Hermitian SHU Ansatz

Consider a structure factor expressed as a superposition of functions possessing *m*‐fold rotational symmetry:

(7) $$S\left(k\right)=\sum_{q\geq 0}\left[A_{q}\left(|k|\right)\cos\left(\mathrm{qm}\theta \left(k\right)\right)+B_{q}\left(|k|\right)\sin\left(\mathrm{qm}\theta \left(k\right)\right)\right].$$

We retain the zeroth (*q* = 0) and first harmonic (*q* = 1) terms while imposing nonnegativity constraint *S*(**k**) ≥ 0 and assuming a common |**k**|*^α^* dependence for SHU [13], where *α* > 0 is the power‐law exponent characterizing the degree of SHU for *S*(|**k**| ≤ *K*) ≈ 0. The result yields the following ansatz of *S*_A_(**k**) and *S*_C_(**k**):

(8) $$\begin{array}{c} S_{A}\left(k\right)∼\frac{1}{2}{\left|k\right|}^{\alpha }\left(1+\frac{1}{2}\left[1+{\left(-1\right)}^{m}\right]\cos\left(m\varphi_{k}\right)\right),\\ S_{C}\left(k\right)∼\frac{1}{2}{\left|k\right|}^{\alpha }\left(\frac{1}{2}\left[1-{\left(-1\right)}^{m}\right]\cos\left(m\varphi_{k}\right)\right). \end{array}$$

The resulting structure factor *S*(|**k**| ≤ *K*) = |**k**|*^α^* [1 + cos(*mφ* **_k_**)]/2 provides an example of scattering responses with *m*‐fold rotational symmetry, as described in Figure 3.

### 4.5 Inverse Design

Under periodic boundary conditions on a square supercell of side length *L*, the set of **k** contributing to the structure factor is restricted to a finite discrete set, *B* = {**k** | **k** = 2*π*(*n_x_*, *n_y_*)/*L*, *n_x_*, *n_y_* ∈ ℤ, 0< |**k**| ≤ *K*}. To optimize point patterns with a target structure factor *S*(**k**), we define the loss function using weighted least‐squares:

(9) $$L\left(r_{1},\cdots ,r_{N}\right)=\sum_{k\in B}\frac{{\left(S\left(k\right)-S_{0}\left(k\right)\right)}^{2}}{{\left|k\right|}^{2}},$$

which assigns larger weights to longer‐wavelength suppression. Its gradient is given by

(10) ∂L∂rp=4∑j=1Nfj2Im∑k∈Bkk2S(k)−S0(k)C*(k)fpe−ik·rp,

where *C* ^*^(**k**) denotes the complex conjugate of the collective coordinate variable. We use the Flatiron Institute Nonuniform Fast Fourier Transform (FINUFFT) for the type‐1 transform to compute collective coordinate variables and the type‐2 transform to evaluate the gradient [29, 30]. Each update direction is obtained using conjugate gradient method with a step size determined by a line search satisfying the Wolfe condition.

### 4.6 Stochastic Band Estimation

To estimate the dispersion relations of our disordered materials—non‐Hermitian SHU—we employ the band unfolding method [34, 35, 36], which extracts the momentum components of the primitive Brillouin zone in the supercell. In this estimation, each realization of disordered materials is defined in a two‐dimensional (2D) supercell, which contains *M* × *M* square primitive cells of period *Λ* = *a*/$\sqrt{2}$ (*M* = 20). For the comparison with the previous study on exceptional contours in PT‐symmetric crystals [33], while maintaining the isotropy of scatterers, we employ cylindrical‐rod scatterers of radius 0.479*Λ* and complex‐valued permittivity *ε* of Re[*ε*] = 12, which are embedded in air. For the uniformly sampled supercell momenta **β**_S_, we calculate eigenstates *ψ_n_* (*m*, **r**) defined in the *m*‐th primitive cell (*m* = 1, 2, …, *M* ^2^) with the intra‐cell coordinate **r** and their corresponding complex eigenfrequencies *ω_n_* for the transverse‐magnetic (TM) mode, by using a finite‐difference frequency‐domain (FDFD) solver [46]. By assigning each **β**_S_ to the primitive momentum **β** *_l_* = **β**_S_ + (2*πl_x_*/(*MΛ*), 2*πl_y_*/(*MΛ*)), folded into [−*π*/*Λ*, + *π*/*Λ*)^2^ with a suitable setting of the integer pair *l* ≜ (*l_x_*, *l_y_*), the spectral weight of mode *n* is computed as

(11) $$P_{n}\left(\beta_{l}\right)=\frac{\int_{{\left[0,\Lambda \right)}^{2}}{\left|\sum_{m}\psi_{n}\left(m,r\right)e^{-i\beta_{l}\cdot R_{m}}\right|}^{2}d^{2}r}{M^{2}\sum_{m}\int_{{\left[0,\Lambda \right)}^{2}}{\left|\psi_{n}\left(m,r\right)\right|}^{2}d^{2}r},$$

where **R** *_m_* is the primitive‐cell position. Following the method in prior studies [34, 35, 36], we apply the following kernel smoothing in estimating the stochastic spectral function:

(12) Aβ,ω=∑n,lPnβlKββ−βlKωω−Re(ωn),

where *K* **_β_** and *K_ω_* are the momentum and frequency Gaussian smoothing kernels, respectively.

## Author Contributions

S.Yu, X.P., and N.P. conceived the idea of generalizing HU and SHU to non‐Hermitian wave physics and supervised the project. S.Youn, G.L., and I.L. carried out the theoretical work and performed the numerical analyses. K.P. and D.H. contributed to discussions on the practical implementation of the proposed design. S.P. contributed to calculating stochastic dispersion bands for SHU materials. All authors contributed to discussions of the results and to the writing of the manuscript.

## Conflicts of Interest

The authors declare no conflicts of interest.

## Supporting information




**Supporting File**: advs77054‐sup‐0001‐SuppMat.pdf.

## Data Availability

The data that support the findings of this study are openly available in zenodo at https://zenodo.org/records/18575032, reference number [47].
