# Supplementary material for: Non‐Hermitian Stealthy Hyperuniformity
Source: Adv Sci (Weinh). 2026 Aug 6;13(49):e77054. doi: 10.1002/advs.77054 (PMC13445321; doi:10.1002/advs.77054)
Supplement: Supplementary file 1 [file ADVS-13-e77054-s001.pdf]

Supplementary Information for "Non-Hermitian stealthy hyperuniformity"

Gitae Lee[1‡], Seungmok Youn[1‡], Ikbeom Lee[1‡], Kunwoo Park[1], Duhwan Hwang[1], Seungkyun Park[1,2,4], Xianji Piao[3§], Namkyoo Park[2†], and Sunkyu Yu[1*]

[1]Intelligent Wave Systems Laboratory, Department of Electrical and Computer Engineering, Seoul National University, Seoul 08826, Korea

[2]Photonic Systems Laboratory, Department of Electrical and Computer Engineering, Seoul National University, Seoul 08826, Korea

[3]Wave Engineering Laboratory, School of Electrical and Computer Engineering, University of Seoul, Seoul 02504, Korea

[4]KAIST InnoCORE PICORE Center, Korea Advanced Institute of Science and Technology, Daejeon, 34141, Korea

E-mail address for correspondence: [§]piao@uos.ac.kr, [†]nkpark@snu.ac.kr, [*]sunkyu.yu@snu.ac.kr

Note S1. Structure factors for non-Hermitian materials

Note S2. Semi-analytical expressions of density fluctuations

Note S3. Loss function and orthogonality of S_A and S_C

Note S4. Non-Hermitian perturbed lattices

Note S5. Full-wave simulations

Note S6. Capturing unidirectionality with two correlated windows

Note S7. Bounds on potentials for the Born approximation

Note S8. Passive realizations

Note S1. Structure factors for non-Hermitian materials

In the weak scattering regime under a far-field measurement condition, the scattering intensity is proportional to the generalized structure factor $S(\mathbf{k})$ which is defined as[1,2]:

$$S(\mathbf{k}) = \frac{1}{V_{\Omega}} \left| \int V_{\mathrm{a}}(\mathbf{r}) e^{-i\mathbf{k}\cdot\mathbf{r}} d\mathbf{r} \right|^2 . \tag{S1}$$

Expanding Eq. (S1) with $V_{\mathrm{a}}(\mathbf{r}) = V_{\mathrm{r}}(\mathbf{r}) + iV_{\mathrm{i}}(\mathbf{r})$ yields:

$$S(\mathbf{k}) = \frac{1}{V_{\Omega}} \left(\left| V_{\mathrm{r}}(\mathbf{k}) \right|^2 + \left| V_{\mathrm{i}}(\mathbf{k}) \right|^2 \right) + \frac{2}{V_{\Omega}} \mathrm{Im}\left[V_{\mathrm{r}}(\mathbf{k}) V_{\mathrm{i}}^{*}(\mathbf{k}) \right], \tag{S2}$$

where $V_{\mathrm{r}}(\mathbf{k})$ and $V_{\mathrm{i}}(\mathbf{k})$ denote the reciprocal-space potentials of $V_{\mathrm{r}}(\mathbf{r})$ and $V_{\mathrm{i}}(\mathbf{r})$, respectively. To analyse finite-size samples in view of the thermodynamic limit, ergodicity requires calculating the statistical average of the samples[3], leading to Eq. (1) in the main text.

Note S2. Semi-analytical expressions of density fluctuations

In this note, we derive semi-analytical expressions for auto- and cross-density fluctuations in statistically homogeneous non-Hermitian materials, providing a comprehensive characterization of their dependence on the window scale R.

Cross-density fluctuation of the real and imaginary potential (σ_C^2)

Based on the real and imaginary potential perturbations in Eq. (4) of Methods, first consider the estimation of the cross-density fluctuation, $\sigma_C^2(R) = \langle N_r N_i \rangle - \langle N_r \rangle \langle N_i \rangle$, which can be written as:

$$\begin{aligned}\sigma_C^{\,2}(R) &= f_r f_i \left\langle \sum_p s_p w^2(\mathbf{r}_p - \mathbf{x}_0; R) \right\rangle + f_r f_i \left\langle \sum_{p \neq q} s_p w(\mathbf{r}_p - \mathbf{x}_0; R) w(\mathbf{r}_q - \mathbf{x}_0; R) \right\rangle \\ &\quad - f_r f_i \left\langle \sum_p w(\mathbf{r}_p - \mathbf{x}_0; R) \right\rangle \left\langle \sum_p s_p w(\mathbf{r}_p - \mathbf{x}_0; R) \right\rangle. \end{aligned} \tag{S3}$$

To evaluate Eq. (S3), we introduce an augmented probability space ($\mathbf{r}$, s), where $s = \pm 1$ labels the sign of the imaginary potential. The first moments appearing in Eq. (S3) are given by

$$\begin{aligned}\left\langle \sum_p w(\mathbf{r}_p - \mathbf{x}_0; R) \right\rangle &= \sum_{s \in \{-1,1\}} \int \rho(\mathbf{r}, s) w(\mathbf{r} - \mathbf{x}_0; R) d\mathbf{r} \\ &= \left(\rho_{-1} + \rho_1 \right) A(R) = \rho A(R), \end{aligned} \tag{S4}$$

$$\begin{aligned}\left\langle \sum_p s_p w(\mathbf{r}_p - \mathbf{x}_0; R) \right\rangle &= \sum_{s \in \{-1,1\}} \int \rho(\mathbf{r}, s) s \cdot w(\mathbf{r} - \mathbf{x}_0; R) d\mathbf{r} \\ &= \left(\rho_1 - \rho_{-1} \right) A(R) = \left\langle s_p \right\rangle \rho A(R), \end{aligned} \tag{S5}$$

which simplify the first and third terms in Eq. (S3) with $w(\mathbf{r} - \mathbf{x}_0; R) = w^2(\mathbf{r} - \mathbf{x}_0; R)$ and the constant number density $\rho_s = \rho(\mathbf{r},s)$ under the statistical homogeneity.

The second term of Eq. (S3) involves the two-particle correlation. From the following Campbell's theorem on the second momentum:

$$\left\langle \sum_{p \neq q} f(\mathbf{r}_p, \mathbf{r}_q) \right\rangle = \sum_{s,s'} \int f(\mathbf{x}, \mathbf{y}) \rho^{(2)}(\mathbf{x}, s, \mathbf{y}, s') d\mathbf{x} d\mathbf{y}, \tag{S6}$$

where $\rho^{(2)}(\mathbf{x},s,\mathbf{y},s')$ denotes the second-order product density in the augmented space—that is, the product of the density values at $\mathbf{x}$ and $\mathbf{y}$ with corresponding s and s'—we obtain

$$\begin{aligned} &\left\langle \sum_{p \neq q} s_p w(\mathbf{r}_p - \mathbf{x}_0; R) w(\mathbf{r}_q - \mathbf{x}_0; R) \right\rangle \\ &= \sum_{s \in \{-1,1\}} s \sum_{s' \in \{-1,1\}} \int w(\mathbf{x}; R) w(\mathbf{y}; R) \rho^{(2)}(\mathbf{x}, s, \mathbf{y}, s') d\mathbf{x} d\mathbf{y}. \end{aligned} \tag{S7}$$

Due to the statistical homogeneity, this quantity can be expressed as

$$\begin{aligned} &\int w(\mathbf{x}; R) w(\mathbf{y}; R) \rho^{(2)}(\mathbf{x}, s, \mathbf{y}, s') d\mathbf{x} d\mathbf{y} \\ &= \rho^2 \int w(\mathbf{x}; R) w(\mathbf{y}; R) g_2(\mathbf{x} - \mathbf{y}, s, s') d\mathbf{x} d\mathbf{y} \\ &= \rho^2 \int w(\mathbf{u}; R) w(\mathbf{u} - \mathbf{r}; R) g_2(\mathbf{r}, s, s') d\mathbf{u} d\mathbf{r}, \end{aligned} \tag{S8}$$

where $g_2(\mathbf{r},s,s')$ is the augmented pair correlation function. For fixed s and s', the spatial integral can be written in terms of the scaled intersection area,

$$\begin{aligned} &\left\langle \sum_{p \neq q} s_p w(\mathbf{r}_p - \mathbf{x}_0; R) w(\mathbf{r}_q - \mathbf{x}_0; R) \right\rangle \\ &= \rho^2 A(R) \int \alpha(\mathbf{r}; R) \sum_{s,s'} s g_2(\mathbf{r}, s, s') d\mathbf{r} \\ &= \rho^2 A(R) \int \alpha(\mathbf{r}; R) \left\{ g_2(\mathbf{r}, 1) - g_2(\mathbf{r}, -1) \right\} d\mathbf{r}, \end{aligned} \tag{S9}$$

where the dependences on the window shape and size are fully captured by the scaled intersection area $\alpha(\mathbf{r}; R)$, as

$$\alpha(\mathbf{r}; R) = \frac{A_2^{\text{int}}(\mathbf{r}; R)}{A(R)}, \quad A_2^{\text{int}}(\mathbf{r}; R) \triangleq \int w(\mathbf{u}; R) w(\mathbf{u} - \mathbf{r}; R) d\mathbf{u}. \tag{S10}$$

We further define $g_2(\mathbf{r},s) \triangleq g_2(\mathbf{r},s,1) + g_2(\mathbf{r},s,-1)$, which encodes correlations as a function of relative position and the sign of the imaginary part at the destination. The cross-density fluctuation then becomes:

$$\frac{\sigma_{\mathrm{C}}{}^2(R)}{\rho A(R)} = f_{\mathrm{r}} f_{\mathrm{i}} \left\langle s_p \right\rangle \{1-\rho A(R)\} + f_{\mathrm{r}} f_{\mathrm{i}} \rho \int \alpha(\mathbf{r};R) \{g_2(\mathbf{r},1) - g_2(\mathbf{r},-1)\} d\mathbf{r}. \tag{S11}$$

Auto-density fluctuation of the real potential ($\sigma_{\mathrm{AR}}{}^2$)

The auto-density fluctuation of real potential, $\sigma_{\mathrm{AR}}{}^2$, is defined by $\langle N_{\mathrm{r}}{}^2\rangle - \langle N_{\mathrm{r}}\rangle^2$, leading to:

$$\begin{aligned} \sigma_{\mathrm{AR}}{}^2(R) = {} & f_{\mathrm{r}}^2 \left\langle \sum_p w^2(\mathbf{r}_p - \mathbf{x}_0; R) \right\rangle + f_{\mathrm{r}}^2 \left\langle \sum_{p \neq q} w(\mathbf{r}_p - \mathbf{x}_0; R) w(\mathbf{r}_q - \mathbf{x}_0; R) \right\rangle \\ & - f_{\mathrm{r}}^2 \left\langle \sum_p w(\mathbf{r}_p - \mathbf{x}_0; R) \right\rangle \left\langle \sum_p w(\mathbf{r}_p - \mathbf{x}_0; R) \right\rangle. \end{aligned} \tag{S12}$$

Because the imaginary components are irrelevant and all real components are identical, the dependence on s and s' can be eliminated by defining $g_2(\mathbf{r}) \triangleq \Sigma_{s,s'} g_2(\mathbf{r},s,s')$. Using the following equation:

$$\left\langle \sum_{p \neq q} w(\mathbf{r}_p - \mathbf{x}_0; R) w(\mathbf{r}_q - \mathbf{x}_0; R) \right\rangle = \rho^2 A(R) \int g_2(\mathbf{r}) \alpha(\mathbf{r}; R) d\mathbf{r}, \tag{S13}$$

which is the direct consequence of the Campbell's theorem of the second momentum, Eq. (S12) reduces to

$$\frac{\sigma_{\mathrm{AR}}{}^2(R)}{\rho A(R)} = f_{\mathrm{r}}^2 \left[1 + \rho \int \left(g_2(\mathbf{r}) - 1 \right) \alpha(\mathbf{r}; R) d\mathbf{r} \right]. \tag{S14}$$

Auto-density fluctuation of the imaginary potential ($\sigma_{\mathrm{AI}}{}^2$)

We consider the auto-density fluctuation of the imaginary potential $\sigma_{\mathrm{AI}}{}^2$, defined as $\langle N_{\mathrm{i}}{}^2\rangle - \langle N_{\mathrm{i}}\rangle^2$, which can be expanded as:

$$
\begin{aligned}
\sigma_{\mathrm{AI}}{}^{2}(R) &= f_{\mathrm{i}}^{2}\left\langle \sum_{p} s_{p}{}^{2} w^{2}(\mathbf{r}_{p}-\mathbf{x}_{0};R)\right\rangle + f_{\mathrm{i}}^{2}\left\langle \sum_{p\neq q} s_{p}s_{q} w(\mathbf{r}_{p}-\mathbf{x}_{0};R) w(\mathbf{r}_{q}-\mathbf{x}_{0};R)\right\rangle \\
&\quad - f_{\mathrm{i}}^{2}\left\langle \sum_{p} s_{p} w(\mathbf{r}_{p}-\mathbf{x}_{0};R)\right\rangle \left\langle \sum_{p} s_{p} w(\mathbf{r}_{p}-\mathbf{x}_{0};R)\right\rangle \\
&= f_{\mathrm{i}}^{2}\rho A(R) + f_{\mathrm{i}}^{2}\left\langle \sum_{p\neq q} s_{p}s_{q} w(\mathbf{r}_{p}-\mathbf{x}_{0};R) w(\mathbf{r}_{q}-\mathbf{x}_{0};R)\right\rangle - f_{\mathrm{i}}^{2}\left\langle s_{p}\right\rangle^{2}\left\{\rho A(R)\right\}^{2}.
\end{aligned}
\tag{S15}
$$

The second term in Eq. (S15) is calculated using $\rho^{(2)}$ as follows:

$$
\left\langle \sum_{p\neq q} s_{p}s_{q} w(\mathbf{r}_{p}-\mathbf{x}_{0};R) w(\mathbf{r}_{q}-\mathbf{x}_{0};R)\right\rangle = \int w(\mathbf{x};R) w(\mathbf{y};R) \sum_{s,s'} ss'\rho^{(2)}(\mathbf{x}-\mathbf{y},s,s') d\mathbf{x} d\mathbf{y}. \tag{S16}
$$

We define the correlation function $c(\mathbf{r})$ as

$$
c(\mathbf{r}) \equiv \sum_{s,s'} ss' g_{2}(\mathbf{r},s,s') = \frac{1}{\rho^{2}} \sum_{s,s'} ss'\rho^{(2)}(\mathbf{r},s,s'), \tag{S17}
$$

which captures correlations in both spatial positions and imaginary components. Using this definition, Eq. (S16) can be rewritten in terms of the scaled intersection area,

$$
\begin{aligned}
&\left\langle \sum_{p\neq q} s_{p}s_{q} w(\mathbf{r}_{p}-\mathbf{x}_{0};R) w(\mathbf{r}_{q}-\mathbf{x}_{0};R)\right\rangle \\
&= \rho^{2}\int w(\mathbf{u};R) w(\mathbf{u}-\mathbf{r};R) c(\mathbf{r}) d\mathbf{u} d\mathbf{r} = \rho^{2} A(R) \int \alpha(\mathbf{r};R) c(\mathbf{r}) d\mathbf{r},
\end{aligned}
\tag{S18}
$$

which leads to the final expression, as follows:

$$
\frac{\sigma^{2}{}_{\mathrm{AI}}(R)}{\rho A(R)} = f_{\mathrm{i}}^{2}\left[1 + \rho\int\left[c(\mathbf{r}) - \left\langle s_{p}\right\rangle^{2}\right]\alpha(\mathbf{r};R) d\mathbf{r}\right]. \tag{S19}
$$

Interpretation of density fluctuations with analytical forms

Figure S1 compares the semi-analytical calculation of density fluctuations, derived from Eq. (S11), (S14), and (S19), with direct numerical measurements obtained in Fig. 2 of the main text. The comparison is shown for three representative classes of point patterns: a PT-symmetric crystal (Fig.

S1a), a disordered non-Hermitian HU material (Fig. S1b), and a non-Hermitian Poisson random material (Fig. S1c). In all cases, the semi-analytical results quantitatively reproduce the numerical measurements over the entire range of window sizes, demonstrating the equivalence of the two estimators and validating the semi-analytical framework developed in this note.

Our semi-analytical framework reveals further design freedom for tailoring the microstructures of non-Hermitian materials by leveraging their cross-density fluctuations. As shown in Eq. (S11), the vanishing of σ_C^2 originates from the balanced numbers of gain and loss particles with $\langle s_p \rangle = 0$ and their identical statistical distributions with $g_2(\mathbf{r},1) = g_2(\mathbf{r},-1)$. From this observation, we present an example of non-Hermitian HU materials that exhibits nonzero cross-density fluctuations at finite R by employing distinct statistical distributions for the gain and loss scatterers (Fig. S1d,e).

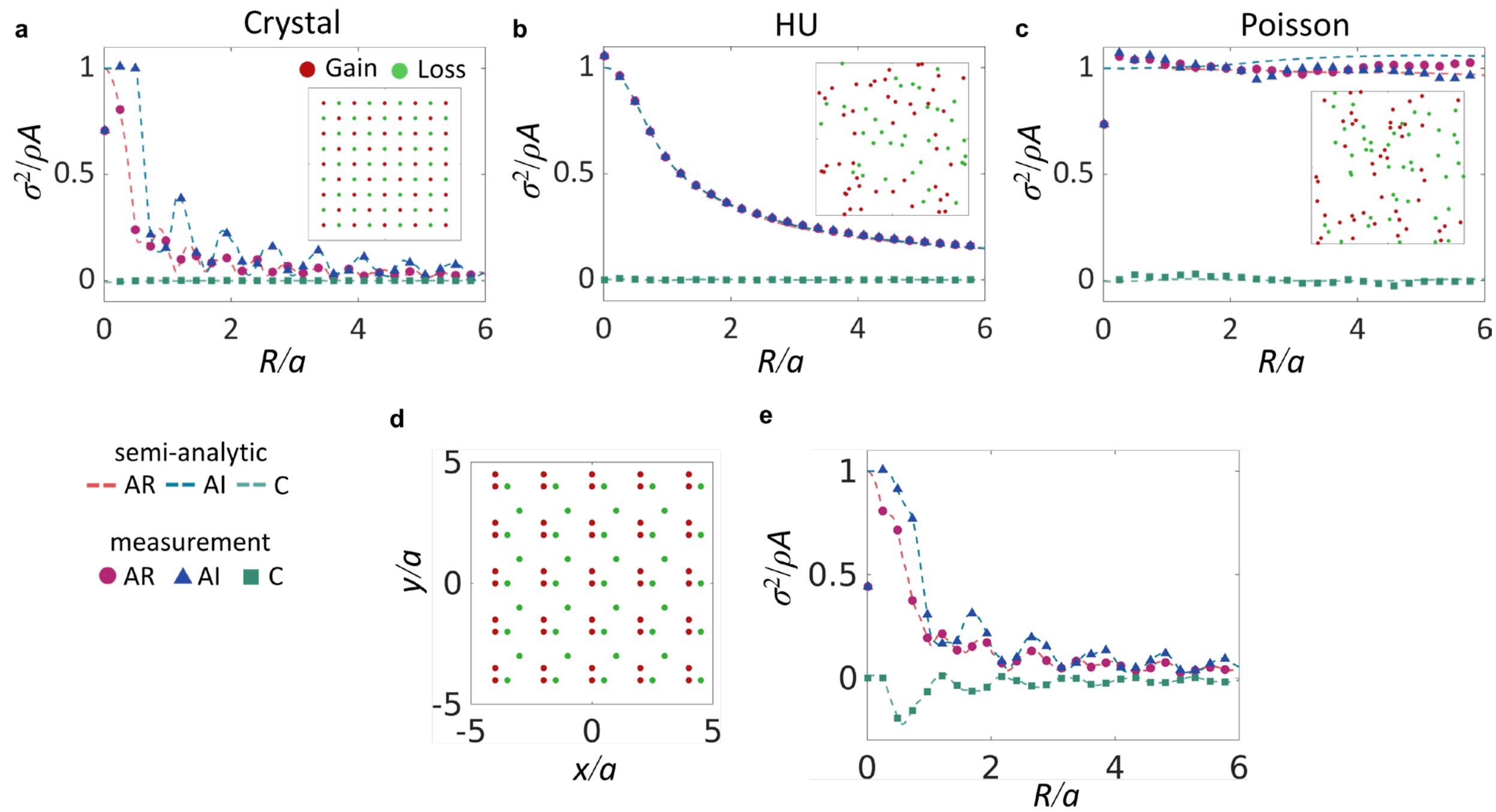

Fig. S1. Semi-analytical density fluctuations in non-Hermitian point patterns. a-c, Comparison between semi-analytical predictions (dashed lines) and numerical measurements (markers) for a square PT-symmetric lattice (**a**), a disordered non-Hermitian HU (**b**), and a non-Hermitian Poisson-random (**c**) material, corresponding to the structures shown in Fig. 2 of the main text. Insets show representative real-space configurations. The excellent agreement across all three classes confirms the validity of the semi-analytical estimator. (**d**) Non-Hermitian HU material with nonzero cross-density at finite R (**e**).

Note S3. Loss function and orthogonality of S_{A} and S_{C}

To generate point patterns with a designed structure factor, we minimize a weighted least-squares objective

$$\mathcal{L} = \sum_{\mathbf{k}\in B} \omega\left(|\mathbf{k}|\right)\left(S(\mathbf{k}) - S_0(\mathbf{k})\right)^2, \tag{S20}$$

using radial weights $\omega(|\mathbf{k}|) = 1/|\mathbf{k}|^2$, design region $B = \{\mathbf{k} \mid \mathbf{k} = 2\pi(n_x,n_y)/L,\ n_x,\ n_y \in \mathbb{Z},\ 0 < |\mathbf{k}| \leq K\}$, and a target structure factor $S_0 = S_{0,\mathrm{A}} + S_{0,\mathrm{C}}$. The corresponding weighted inner product is defined as:

$$\left\langle f, g\right\rangle_{\omega} \equiv \sum_{\mathbf{k}\in B} \omega\left(|\mathbf{k}|\right) f(\mathbf{k}) g(\mathbf{k}). \tag{S21}$$

By pairing $(\mathbf{k}, -\mathbf{k})$ and using the relationships, $S_{\mathrm{A}}(-\mathbf{k}) = S_{\mathrm{A}}(\mathbf{k})$ and $S_{\mathrm{C}}(-\mathbf{k}) = -S_{\mathrm{C}}(\mathbf{k})$, we can utilize $\langle S_{\mathrm{A}}, S_{\mathrm{C}}\rangle_{\omega} = 0$. Consequently, the loss function separates additively as

$$\mathcal{L} = \left\| S_{\mathrm{A}} - S_{0,\mathrm{A}} \right\|^2 + \left\| S_{\mathrm{C}} - S_{0,\mathrm{C}} \right\|^2, \tag{S22}$$

where $\|f\|$ denotes the weighted norm defined by $\langle f, f\rangle_{\omega}$. This orthogonality guarantees independent convergence of S_{A} and S_{C} toward their respective target ansatzes, $S_{0,\mathrm{A}}$ and $S_{0,\mathrm{C}}$, respectively.

Note S4. Non-Hermitian perturbed lattices

Materials with unidirectional scattering properties at long wavelengths can also be realized by perturbing non-Hermitian lattices (Fig. S2). Depending on the propagation direction, the resulting materials exhibit an angle-dependent unsuppressed region of the structure factor near the origin of reciprocal space. These materials can be regarded as the defected-crystalline counterparts of non-Hermitian disordered SHU systems.

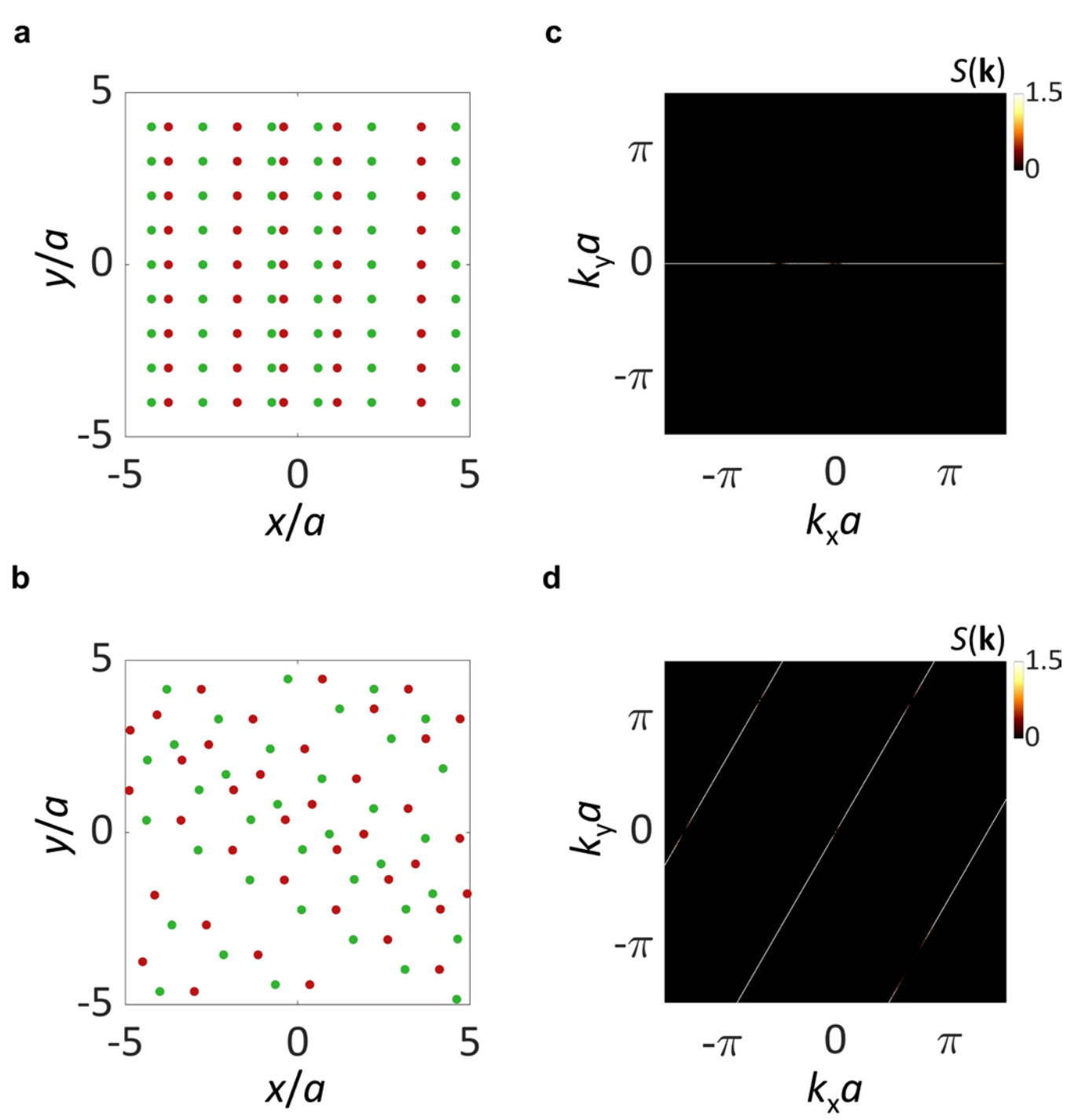

Fig. S2. Non-Hermitian perturbed lattices. a,b, Realizations of perturbed non-Hermitian lattices, where perturbations are applied on the square lattice (**a**) and a hexagonal lattice (**b**), which correspond to the crystalline structures shown in Fig. 3f and Fig. 3g of the main text, respectively. **c-d,** Corresponding structure factors of perturbed square (**c**) and hexagonal (**d**) lattices.

Note S5. Full-wave simulations

We validate our analysis using full-wave finite-difference time-domain (FDTD) simulations performed with Tidy3D[4]. The realization generated in a dimensionless square domain $[0, L]^2$ is mapped to physical coordinates through a scale factor $\xi = K\lambda_0/(2\pi)$, where $\lambda_0 = 0.5\ \mu$m denotes the free-space wavelength. This scaling ensures that the accessible momentum-transfer grid, expressed in units of $k_0=2\pi/\lambda_0$, spans $\mathbf{k}/k_0 \in [-2, 2]^2$. The physical size of the scattering region becomes $L_{SC} = L\xi$.

Each scatterer is modelled as a cylindrical rod of radius $r_{rod} = \lambda_0/45$, positioned at the optimized locations $\mathbf{r}_p$. The scatterers possess a complex refractive index $n_p = 1 + \Delta n + i\kappa_p$, with $\Delta n = 0.05$ and $\kappa_p \in \{0.05, -0.05\}$. The background medium is air ($n = 1$). We employ a uniform Cartesian grid with a spatial resolution of $r_{rod}/7$. The structure is examined using a plane-wave total-field/scattered-field (TFSF) harmonic source with frequency $f_0 = c/\lambda_0$. The polarization is set to the transverse-electric mode.

The azimuthal angle of incidence θ_{inc} is swept from 0° to 357° in steps of 3°. Four rectangular field monitors (top, bottom, left, and right) are placed outside the TFSF boundary at the distance of $0.2\lambda_0$. The recorded near fields are projected onto a far-field angular ring using Tidy3D's angle projector[4], sampling observation angles $\theta \in [0,2\pi]$ with a resolution of 3°. Each simulation runs for a total time of $5L_{SC}/c$, which is sufficient to ensure convergence.

The projected far-field intensity measured at angle θ for a plane-wave incidence from direction θ_{inc}, denoted as $I(\theta;\ \theta_{inc})$, is normalized by the scattering intensity $I_0(\theta)$—defined as the sum of the scattering intensities of individual gain and loss—to obtain the structure factor:

$$S(\mathbf{k}) \sim \frac{I(\theta;\theta_{inc})}{I_0(\theta;\theta_{inc})}. \tag{S23}$$

Finally, we decompose the simulated structure factor into its auto- and cross- components: $S_A(\mathbf{k}) = [S(\mathbf{k}) + S(-\mathbf{k})]/2$, $S_C(\mathbf{k}) = [S(\mathbf{k}) - S(-\mathbf{k})]/2$. For an inversely designed sample with $\alpha = 0.2$, consisting of 2000 gain and 2000 loss scatterers, we truncate a circular region in real space and obtain $S(\mathbf{k})$ (Fig. S3a,b), $S_A(\mathbf{k})$ (Fig. S3c,d), and $S_C(\mathbf{k})$ (Fig. S3e,f). Structure factors of $m = 2$ (Fig. S3a,c,e) and $m = 3$ (Fig. S3b,d,f) show clear 2- and 3-fold rotational symmetry of suppression region, respectively, as demonstrated in the main text.

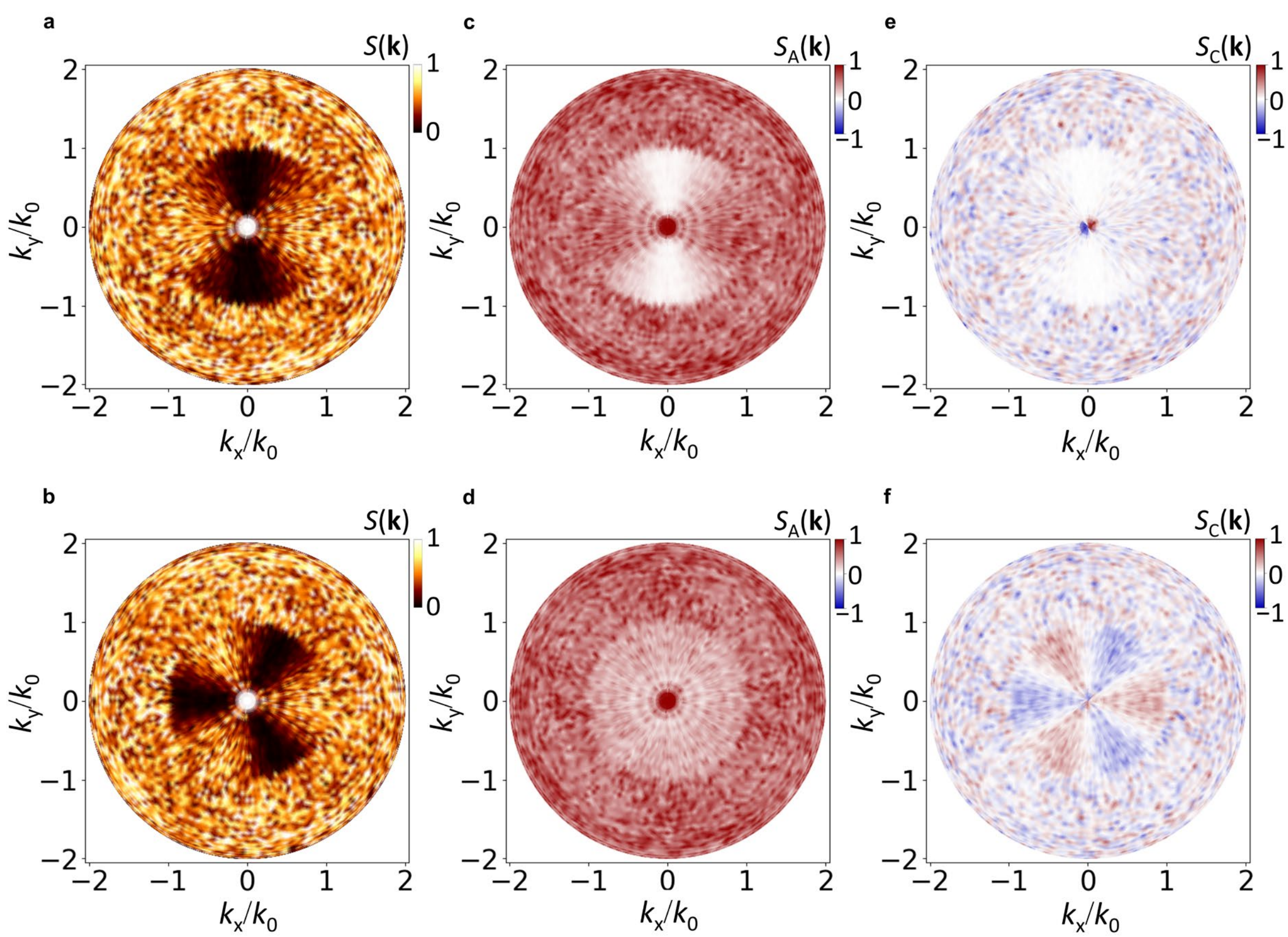

Fig. S3. Structure factors obtained with full-wave analysis. Total structure factors $S(\mathbf{k})$ for $m = 2$ (**a**) and $m = 3$ (**b**). Corresponding $S_A(\mathbf{k})$ (**c**,**d**) and $S_C(\mathbf{k})$ (**e**,**f**). To ensure statistical robustness, 5 independent sample realizations are averaged.

Note S6. Capturing unidirectionality with two correlated windows

The odd component of the structure factor, $S_C(\mathbf{k})$, which underlies unidirectional scattering, can be expressed as the Fourier transform of the real-imaginary correlation function $\rho_{ri,2}(\mathbf{r})$:

$$\begin{gathered}\rho_{\mathrm{ri},2}(\mathbf{r}) \triangleq \int V_{\mathrm{r}}(\mathbf{r}')V_{\mathrm{i}}(\mathbf{r}'+\mathbf{r})d\mathbf{r}', \\ S_{\mathrm{C}}(\mathbf{k}) \propto \int\left(\rho_{\mathrm{ri},2}(\mathbf{r})-\rho_{\mathrm{ri},2}(-\mathbf{r})\right)e^{i\mathbf{k}\cdot\mathbf{r}}d\mathbf{r} = 2\int \mathcal{O}\left(\rho_{\mathrm{ri},2}(\mathbf{r})\right)e^{i\mathbf{k}\cdot\mathbf{r}}d\mathbf{r},\end{gathered} \tag{S24}$$

where $\mathcal{O}\{\cdot\}$ denotes the odd part of a function. Because $S_C(\mathbf{k})$ is directly related to the odd component of $\rho_{ri,2}(\mathbf{r})$, conventional sampling schemes employing a single window fail to capture it due to the even-functional property of the associated kernel:

$$\left\langle N_{\mathrm{r}}(\mathbf{r};\mathbf{R})N_{\mathrm{i}}(\mathbf{r};\mathbf{R})\right\rangle = A(\mathbf{R})\int d\mathbf{r}\rho_{\mathrm{ri},2}(\mathbf{r})\alpha(\mathbf{r};\mathbf{R}), \tag{S25}$$

where $\mathbf{R}$ specifies the orientation and size of a window of given shape, $N_r(\mathbf{r};\mathbf{R})$ and $N_i(\mathbf{r};\mathbf{R})$ denote the net real and imaginary potential perturbations within a window placed at $\mathbf{r}$, respectively, and $\alpha(\mathbf{r};\mathbf{R})$ represents the scaled intersection volume of windows with volume $A(\mathbf{R})$. Because $\alpha(\mathbf{r};\mathbf{R}) = \alpha(-\mathbf{r};\mathbf{R})$, the odd component of $\rho_{ri,2}(\mathbf{r})$ cannot be detected. To overcome this limitation, we introduce two distinct but spatially correlated windows in each trial, which generally breaks the even-functional property of $\alpha(\mathbf{r};\mathbf{R})$ and allows odd contributions:

$$\begin{gathered}\left\langle N_{1,\mathrm{r}}(\mathbf{r};\mathbf{R})N_{2,\mathrm{i}}(\mathbf{r};\mathbf{R})\right\rangle = A(\mathbf{R})\int d\mathbf{r}\rho_{\mathrm{ri},2}(\mathbf{r})\alpha_{12}(\mathbf{r};\mathbf{R}), \\ \alpha_{12}(\mathbf{r};\mathbf{R}) = \frac{1}{A(\mathbf{R})}\int d\mathbf{r}'w_1(\mathbf{r}';\mathbf{R})w_2(\mathbf{r}'+\mathbf{r};\mathbf{R}).\end{gathered} \tag{S26}$$

In general, the resulting cross-kernel, $\alpha_{12}(\mathbf{r};\mathbf{R})$, is not an even function, even for circular windows. Specifically, we employ two circular windows of the identical radius a, the characteristic length, whose centres are separated by relative displacement $\mathbf{R}_c = r(\hat{\mathbf{x}}\cos\varphi + \hat{\mathbf{y}}\sin\varphi)$. The first window only measures the net real potential perturbation within it, denoted as $N_{1,r}$, while the second window measures only the net imaginary potential perturbation, denoted as $N_{2,i}$. The odd-part of real-

imaginary correlation function is then captured by trial measurements while varying the direction and norm of $\mathbf{R}_c$.

Note S7. Bounds on potentials for the Born approximation

In this note, we provide a simple empirical bound on the complex-valued potential for the validity of the first-order Born approximation used in the main text. Iterating the Lippmann-Schwinger equation in the main text generates the Born series $\psi = \psi_{\text{in}} + \psi^{(1)} + \psi^{(2)} + \cdots$, where $\psi^{(n)}$ describes the n-th order scattering by the potential $V_{\text{a}}(\mathbf{r})$. The first-order approximation—which underlies the structure factor $S(\mathbf{k})$ in Eq. (1) of the main text—is justified when the second-order term is subdominant to the first, that is, $|\psi^{(2)}| << |\psi^{(1)}|$. Because the second-order scattering is obtained recursively, as

$$\psi^{(2)}(\mathbf{r}) = \frac{1}{4\pi}\int G(\mathbf{r},\mathbf{r}';\mathbf{k}_{\text{S}})V_{\text{a}}(\mathbf{r}')\psi^{(1)}(\mathbf{r}')d\mathbf{r}', \tag{S27}$$

it is useful to introduce the following practical dimensionless parameter:

$$q \triangleq \max_{\mathbf{r}}\left(\frac{1}{4\pi}\int \left|G(\mathbf{r},\mathbf{r}';\mathbf{k}_{\text{S}})\right|\left|V_{\text{a}}(\mathbf{r}')\right|d\mathbf{r}'\right), \tag{S28}$$

which bounds the relative magnitude of the second-order term $|\psi^{(2)}|/|\psi^{(1)}|$ as $|\psi^{(2)}(\mathbf{r})| \leq q \times \sup(|\psi^{(1)}|)$ for all $\mathbf{r}$.

Using the two-dimensional Green's function $G(\mathbf{r},\mathbf{r}';\mathbf{k}_{\text{S}}) = i\pi H_0^{(1)}(k|\mathbf{r} - \mathbf{r}'|)$ together with the small-argument behaviour of the Hankel function, $|H_0^{(1)}(z)| \sim \ln(1/|z|)$ for $|z| << 1$, where $|\mathbf{k}_{\text{I}}| = |\mathbf{k}_{\text{S}}| = k$, the practical Born criterion for small scatterers using $q << 1$ becomes

$$\max\left[\left|V_{\text{a}}(\mathbf{r})\right|\right] r_{\text{S}}^2 \ln\left(\frac{1}{kr_{\text{S}}}\right) \ll 1, \qquad (kr_{\text{S}} \ll 1) \tag{S29}$$

where r_{S} denotes the characteristic radius of a scatterer. On the other hand, for large scatterers, the necessity of small phase accumulation across a single scatterer yields

$$\frac{\max\left[\left|V_{\text{a}}(\mathbf{r})\right|\right] r_{\text{S}}}{k} \ll 1. \qquad (kr_{\text{S}} \geq 1) \tag{S30}$$

Because the background Green's function is that of the Hermitian bias medium, the bounds in Eqs. (S29) and (S30) depend on the non-Hermitian potential only through the modulus $|V_a|$. Therefore, the Hermitian ($\mathrm{Re}[V_a]$) and anti-Hermitian ($\mathrm{Im}[V_a]$) parts of the potential are constrained on an equal footing within the validity bound. Moreover, because the ensemble-averaged imaginary potential vanishes for the balanced gain-loss ensembles employed in the main text, cumulative amplification or attenuation of the incident wave cancels at leading order.

Note S8. Passive realizations

Regarding practical realizations, we implement non-Hermitian SHU materials on a passive platform, using low- and high-loss scatterers without optical gain, analogous to passive realizations of PT symmetry under a gauge transformation[5]. In Note S5, the ratio between the complex-valued perturbed potentials of the gain ($\Delta\varepsilon_-$) and loss ($\Delta\varepsilon_+$) scatterers, defined using $n_p^2 = (1 + \Delta n + i\kappa_p)^2$, is given by $\Delta\varepsilon_+/\Delta\varepsilon_- = -0.05 + i$. For the passive implementation, we approximately preserve this ratio while maintaining positive imaginary parts of the scatterer permittivities. As an example, we set $\Delta\varepsilon_\pm = 0.03 \times \exp(i(1/3 \pm 1/4)\pi)$, yielding $\Delta\varepsilon_+/\Delta\varepsilon_- = i$, for a lossless background medium $\varepsilon_\mathrm{b} = n_\mathrm{b}^2 = 2.25$.

Figure S4 shows the full-wave estimation of the structure factor. The rod radius is set to $r_\mathrm{rod} = \lambda_0/(45n_\mathrm{b})$ with a grid resolution of $r_\mathrm{rod}/7$, the same as those used in Note S5. Analogous to the conservation of exceptional-point dynamics in passive PT symmetry[5], passive non-Hermitian SHU materials also maintain the characteristic distributions of the structure factors $S(\mathbf{k})$, $S_\mathrm{A}(\mathbf{k})$, and $S_\mathrm{C}(\mathbf{k})$ (Fig. S4).

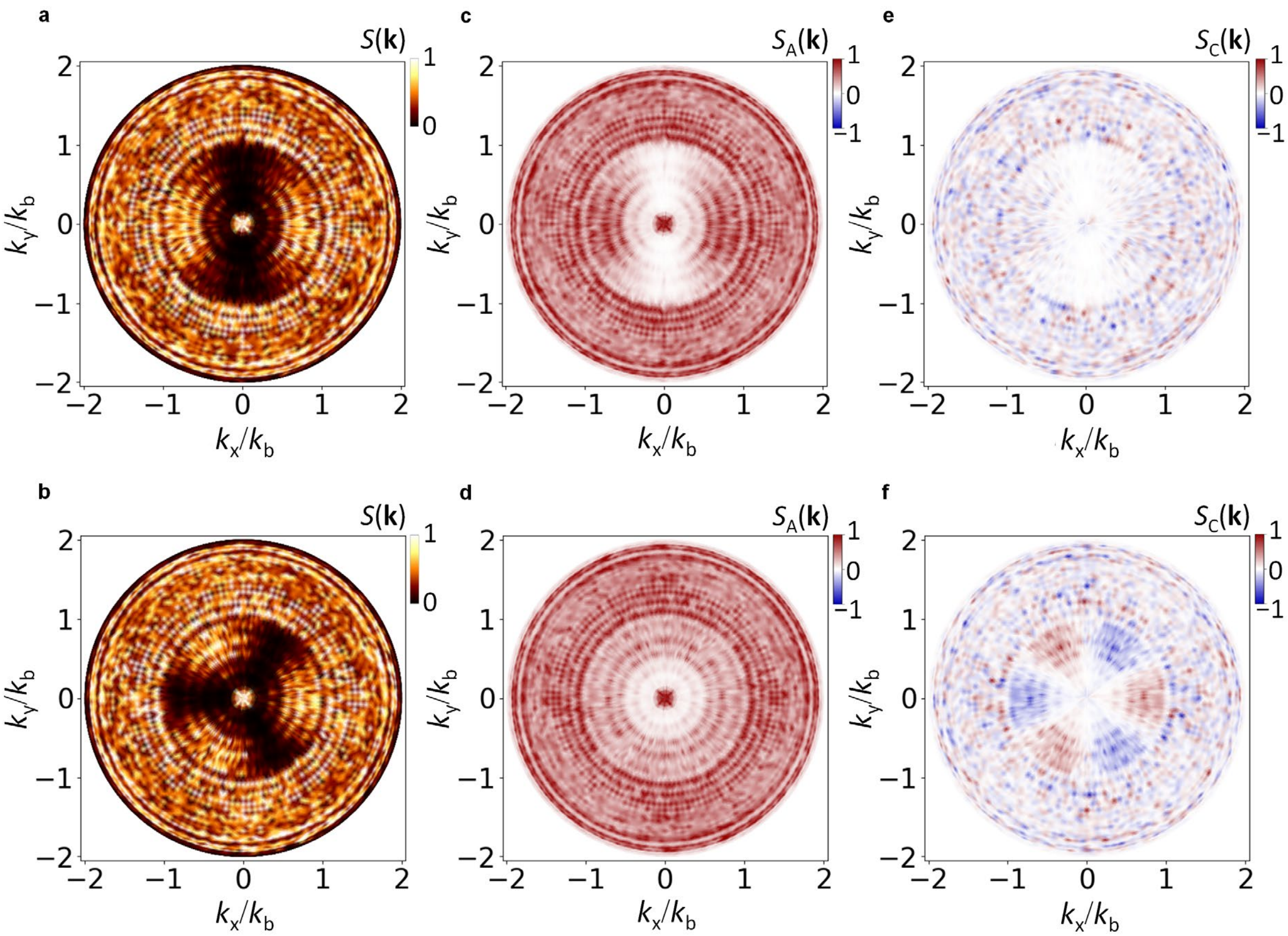

Supplementary Figure S4. Structure factors of passive non-Hermitian SHU materials. Total structure factors $S(\mathbf{k})$ for m = 2 (**a**) and m = 3 (**b**). Corresponding $S_A(\mathbf{k})$ (**c**,**d**) and $S_C(\mathbf{k})$ (**e**,**f**). To ensure statistical robustness, 5 independent sample realizations are averaged. $k_b = n_b k_0$.

References

1. Gonis, A. & Butler, W. H. *Multiple scattering in solids* (Springer Science & Business Media, 1999).
2. Yu, S. Evolving scattering networks for engineering disorder. *Nat. Comput. Sci.* **3**, 128-138 (2023).
3. Torquato, S. *Random Heterogeneous Materials: Microstructure and Macroscopic Properties* (Springer New York, NY, 2002).
4. *Tidy3D, the full-wave simulator employing the finite-difference time-domain (FDTD) method, developed by Flexcompute, Inc.* https://www.flexcompute.com/tidy3d/solver/ *(2024).*
5. Guo, A., Salamo, G. J., Duchesne, D., Morandotti, R., Volatier-Ravat, M., Aimez, V., Siviloglou, G. A. & Christodoulides, D. N. Observation of PT-symmetry breaking in complex optical potentials. *Phys. Rev. Lett.* **103**, 093902 (2009).
